# Pulmonary Reserve and Respiratory Recovery After Cardiac Surgery: Insights from Preoperative Spirometric Phenotyping

**DOI:** 10.3390/biomedicines14081799

**Published:** 2026-08-10

**Authors:** Gabriela Mara, Andrei Raul Manzur, Ana Lascu, Raluca Elisabeta Staicu, Alina Mirela Popa, Loredana Neli Gligor, Andreea-Roxana Florescu, Claudia Borza, Stefan Mihaicuta

**Affiliations:** 1 Faculty of Medicine, Vasile Goldis Western University of Arad, 310414 Arad, Romania; mara.gabriela@uvvg.ro; 2 Multidisciplinary Doctoral School, Vasile Goldiș Western University of Arad, 310414 Arad, Romania; 3 Doctoral School of Medicine and Pharmacy, “Victor Babeș” University of Medicine and Pharmacy Timisoara, Eftimie Murgu Square No. 2, 300041 Timisoara, Romania; raluca.staicu@umft.ro (R.E.S.); alina-mirela.popa@umft.ro (A.M.P.); gligorloredananeli@gmail.com (L.N.G.); andreea.florescu@umft.ro (A.-R.F.); 4 Department of Functional Sciences, Discipline of Pathophysiology, Faculty of Medicine, “Victor Babeș” University of Medicine and Pharmacy, Eftimie Murgu Sq. No. 2, 300041 Timisoara, Romania; borza.claudia@umft.ro; 5 Centre for Translational Research and Systems Medicine, “Victor Babeș” University of Medicine and Pharmacy of Timișoara, Eftimie Murgu Square No. 2, 300041 Timisoara, Romania; 6 Clinic for Cardiovascular Surgery, Institute for Cardiovascular Diseases Timisoara, 300310 Timisoara, Romania; 7 Center for Research and Innovation in Precision Medicine of Respiratory Diseases, Department of Pulmonology, “Victor Babeș” University of Medicine and Pharmacy Timisoara, Eftimie Murgu Square No. 2, 300041 Timisoara, Romania; stefan.mihaicuta@umft.ro

**Keywords:** cardiac surgery, spirometry, pulmonary reserve, restrictive spirometric pattern, mechanical ventilation, ventilator liberation, respiratory recovery, Ventilatory Burden Index, cardiopulmonary reserve, perioperative risk stratification

## Abstract

**Background**: Postoperative respiratory dysfunction remains a frequent complication after cardiac surgery, yet the influence of preoperative pulmonary reserve on postoperative respiratory recovery remains incompletely understood. We investigated whether preoperative spirometric phenotype was associated with postoperative ventilatory burden following cardiac surgery. **Methods**: We performed a secondary analysis of a prospective cohort of adults undergoing elective cardiac surgery with cardiopulmonary bypass. Preoperative spirometry was used to classify patients as having normal spirometry, a restrictive spirometric pattern, or an obstructive spirometric pattern according to ERS/ATS recommendations. The primary endpoint was VBI, a study-specific, unweighted composite defined as the cumulative duration of IPPV, SIMV, invasive CPAP, and post-extubation CPAP/AIRVO support. Univariable and multivariable linear regression analyses were performed to evaluate variables associated with postoperative VBI. **Results**: A total of 129 patients were included. Preoperative spirometry was normal in 82 patients (63.6%), demonstrated a restrictive spirometric pattern in 20 (15.5%), and an obstructive spirometric pattern in 25 (19.4%). VBI did not differ significantly among spirometric phenotypes (*p* = 0.443). In the multivariable analysis, preoperative spirometric phenotype was not significantly associated with VBI. Older age was associated with higher VBI (β = 0.0095, *p* = 0.015), whereas higher left ventricular ejection fraction was associated with lower VBI (β = −0.0161, *p* = 0.032). Exploratory analyses demonstrated a numerical tendency toward longer invasive respiratory-support duration among patients with a moderate restrictive spirometric pattern. **Conclusions**: Broad preoperative spirometric phenotype was not significantly associated with cumulative postoperative respiratory-support burden. Exploratory findings should be regarded as hypothesis-generating. Larger prospective studies are needed to determine whether restrictive spirometric abnormalities are associated with delayed liberation from invasive mechanical ventilation.

## 1. Introduction

Postoperative respiratory dysfunction remains one of the most common challenges following cardiac surgery. Despite advances in perioperative management, many patients require prolonged respiratory support due to the combined effects of median sternotomy, cardiopulmonary bypass, postoperative pain, diaphragmatic dysfunction, fluid shifts, and transient impairment of cardiopulmonary performance [1,2]. Delayed respiratory recovery is associated with increased intensive care utilization, prolonged hospitalization, higher healthcare costs, and worse postoperative outcomes, highlighting the importance of identifying preoperative factors that may influence postoperative ventilatory trajectories [3,4].

Pulmonary function represents an important component of physiological reserve. Spirometry provides a standardized, readily available assessment of ventilatory function and is widely used to identify obstructive ventilatory abnormalities and restrictive spirometric patterns before surgery [5,6,7]. Previous studies have reported associations between impaired pulmonary function and adverse postoperative outcomes, including prolonged mechanical ventilation, pulmonary complications, and extended intensive care unit stays. Nevertheless, the available literature remains heterogeneous, with most investigations focusing on individual spirometric parameters or isolated postoperative outcomes rather than the cumulative burden of respiratory support required throughout postoperative recovery [8,9].

Among spirometric abnormalities, a restrictive spirometric pattern may be particularly relevant in the perioperative setting. Unlike obstructive abnormalities, which primarily affect expiratory airflow, a restrictive spirometric pattern suggests reduced ventilatory capacity and may reflect reduced pulmonary reserve, although confirmation of true restrictive ventilatory impairment requires lung-volume measurements beyond spirometry alone [10,11]. Following cardiac surgery, respiratory-system performance is challenged by postoperative atelectasis, impaired lung compliance, diaphragmatic dysfunction, increased respiratory workload, and systemic inflammatory responses associated with cardiopulmonary bypass. Consequently, patients demonstrating a restrictive spirometric pattern before surgery may be more vulnerable to delayed liberation from mechanical ventilation. Nevertheless, the relationship between preoperative spirometric phenotype and postoperative respiratory recovery remains incompletely understood [12,13,14].

To address this knowledge gap, we performed a secondary analysis of a prospective cohort of patients undergoing elective cardiac surgery. We evaluated whether preoperative spirometric phenotype was associated with postoperative respiratory recovery using the Ventilatory Burden Index (VBI), a study-specific composite measure designed to quantify the cumulative duration of both invasive mechanical ventilation and clinically indicated post-extubation respiratory support, thereby capturing the overall postoperative respiratory-support trajectory rather than relying on a single recovery milestone such as extubation time. In addition to the primary composite endpoint, we examined the individual components of respiratory support and explored the influence of spirometric severity categories on postoperative ventilatory outcomes. We hypothesized that a preoperative restrictive spirometric pattern would be associated with greater postoperative respiratory burden and prolonged respiratory-support requirements compared with normal spirometry or obstructive ventilatory abnormalities.

## 2. Materials and Methods

### 2.1. Study Design and Ethical Approval

This study was conducted and reported in accordance with the STROBE (Strengthening the Reporting of Observational Studies in Epidemiology) guidelines for observational research [15]. The present investigation represents a secondary analysis of a prospectively collected cohort of adult patients undergoing elective cardiac surgery.

The parent cohort was assembled between 2022 and 2025 at the Institute of Cardiovascular Diseases, Timișoara (Institutul de Boli Cardiovasculare Timișoara, IBCVT), a tertiary cardiac surgical center in western Romania, in collaboration with the Department of Pulmonology of the “Victor Babeș” Clinical Hospital for Infectious Diseases and Pulmonology, Timișoara.

The study was approved by the Scientific Research Ethics Committee of the Victor Babeș University of Medicine and Pharmacy, Timișoara; (approval number 101/19 December 2022, revised in 2025). All procedures complied with the principles of the Declaration of Helsinki, and written informed consent was obtained from all participants prior to enrollment.

The objective of the present analysis was to evaluate the association between preoperative spirometric phenotype and postoperative respiratory recovery, assessed using the Ventilatory Burden Index (VBI) and its individual respiratory-support components.

### 2.2. Study Cohort and Eligibility Criteria

This study represents a secondary analysis of a prospectively collected cohort of adult patients undergoing elective cardiac surgery [16]. The parent cohort included 142 patients who underwent preoperative cardiorespiratory polygraphy, spirometric assessment, and standardized postoperative ventilatory monitoring.

For the present analysis, patients were eligible if they fulfilled all of the following criteria: age ≥ 18 years; elective conventional cardiac surgery performed with cardiopulmonary bypass; available preoperative spirometry; complete perioperative surgical timing data, including cardiopulmonary bypass time, ischemic time, and reperfusion time; and complete postoperative ventilatory trajectory data, including invasive ventilation phases and post-extubation respiratory support.

Patients were excluded if they underwent off-pump surgery, minimally invasive or thoracoscopic procedures, isolated congenital defect repair, procedures requiring mechanical circulatory support, or highly atypical surgical trajectories that could substantially alter the standard postoperative respiratory recovery pathway.

**Figure 1:**
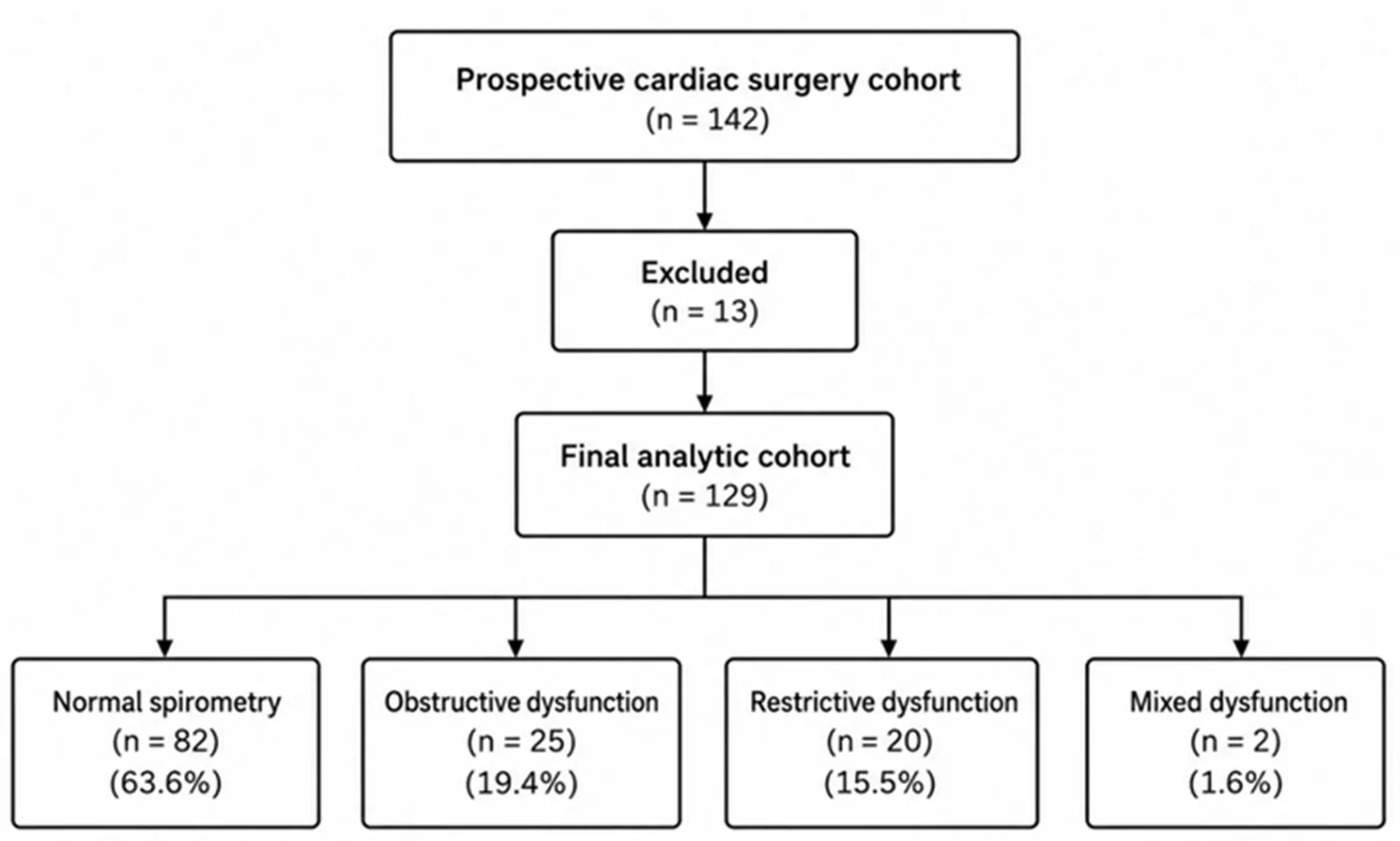
Study flowchart of patient selection and spirometric phenotype classification. Of the 142 patients enrolled in the prospective cardiac surgery cohort, 13 were excluded according to the predefined eligibility criteria, resulting in a final analytic cohort of 129 patients. Preoperative spirometry demonstrated normal findings in 82 patients, obstructive spirometric patterns in 25 patients, restrictive spirometric pattern in 20 patients, and mixed spirometric patterns in 2 patients. Patients with mixed spirometric patterns were excluded from phenotype-based comparative analyses because of insufficient sample size.

After application of the eligibility criteria, 13 patients were excluded from the original cohort of 142 patients, resulting in a final analytic cohort of 129 patients (Figure 1). Patients with incomplete perioperative or postoperative respiratory-support data were excluded from the present analysis. Consequently, the final analytical dataset contained no missing values, no imputation procedures were required, and all analyses were performed using complete-case analysis.

### 2.3. Preoperative Spirometric Assessment and Phenotype Classification

All patients underwent standardized preoperative spirometry within 24–48 h before surgery using the SpiroLab New system (MIR—Medical International Research, Rome, Italy) in accordance with current American Thoracic Society/European Respiratory Society (ATS/ERS) recommendations [17,18].

The recorded spirometric parameters included forced expiratory volume in one second (FEV_1_), forced vital capacity (FVC), and the FEV_1_/FVC ratio. Spirometric phenotypes were classified according to contemporary European Respiratory Society recommendations [18].

Patients were categorized as having normal spirometry, an obstructive spirometric pattern, or a restrictive spirometric pattern based on spirometric interpretation. An obstructive spirometric pattern was defined by an FEV_1_/FVC ratio below the lower limit of normal. A restrictive spirometric pattern was defined by a preserved FEV_1_/FVC ratio in combination with a reduced FVC. Because static lung volumes were not measured, this classification represents a restrictive spirometric pattern suggestive of restrictive ventilatory impairment rather than confirmation of true restrictive ventilatory impairment, which requires demonstration of reduced total lung capacity.

For exploratory analyses, patients were additionally stratified according to spirometric severity using guideline-based categories derived from percent-predicted spirometric values.

### 2.4. Perioperative Data Collection

Demographic, clinical, echocardiographic, and operative variables were prospectively collected. Baseline variables included age, sex, body mass index, smoking status, diabetes mellitus, arterial hypertension, and left ventricular ejection fraction.

Operative variables included surgery type, cardiopulmonary bypass duration, aortic cross-clamp (ischemic) time, and reperfusion time. Surgical procedures were categorized as isolated coronary artery bypass grafting, isolated valve surgery, or combined/complex cardiac procedures.

### 2.5. Postoperative Respiratory Support and Ventilatory Burden Index

Postoperative respiratory-support data were prospectively collected for all patients throughout their intensive care unit stay using standardized institutional monitoring protocols.

The invasive ventilation phase comprised intermittent positive-pressure ventilation (IPPV), synchronized intermittent mandatory ventilation (SIMV), and invasive continuous positive airway pressure (CPAP) delivered via the endotracheal tube before successful extubation. Following extubation, patients received postoperative respiratory support according to institutional protocols, including non-invasive CPAP or high-flow nasal oxygen therapy using the AIRVO™ 2 system (Fisher & Paykel Healthcare, Auckland, New Zealand), when clinically indicated.

The primary respiratory outcome was the Ventilatory Burden Index (VBI), a study-specific composite endpoint developed to quantify the cumulative duration of postoperative respiratory support. Unlike conventional endpoints such as extubation time or invasive ventilation duration alone, VBI was designed to capture the entire postoperative respiratory-support trajectory by integrating both invasive and clinically indicated post-extubation respiratory support.

VBI was calculated as:

VBI (hours) = IPPV duration + SIMV duration + invasive CPAP duration + post-extubation CPAP/AIRVO duration

where all components were expressed in hours and contributed equally to the composite score. No modality-specific weighting factors were applied. Because VBI represents a study-specific composite measure intended to quantify cumulative respiratory-support duration rather than support intensity, its individual components were also analyzed separately.

Secondary respiratory outcomes included invasive respiratory-support duration (defined as the cumulative duration of IPPV, SIMV, and invasive CPAP prior to extubation), the requirement for post-extubation CPAP/AIRVO support, and the duration of post-extubation respiratory support.

### 2.6. Clinical Outcomes

The primary study endpoint was postoperative Ventilatory Burden Index.

Secondary endpoints included individual components of postoperative respiratory support, including invasive respiratory-support duration, post-extubation CPAP/AIRVO requirement, and post-extubation CPAP/AIRVO duration.

Additional exploratory analyses examined respiratory outcomes according to spirometric severity status and individual ventilation-component trajectories.

### 2.7. Bias Minimization

Selection bias was minimized through consecutive recruitment of eligible patients undergoing elective cardiac surgery during the study period according to predefined inclusion and exclusion criteria. Spirometric testing, perioperative data collection, and postoperative respiratory-support assessment followed standardized institutional protocols. Respiratory outcomes were prospectively recorded using objective clinical measurements, thereby reducing the risk of differential outcome assessment. Statistical analyses were conducted using predefined outcome definitions and standardized analytical procedures.

### 2.8. Statistical Analysis

Statistical analyses were performed using R version 4.4.2 and Python version 3.12 using the pandas, SciPy, statsmodels and matplotlib packages. Following application of the eligibility criteria, the analytical dataset contained no missing values; therefore, all analyses were performed using complete-case analysis without imputation.

Continuous variables were assessed for normality using visual inspection of histograms and Q–Q plots, supplemented by the Shapiro–Wilk test. Normally distributed variables are presented as mean ± standard deviation, whereas non-normally distributed variables are reported as median and interquartile range. Categorical variables are presented as frequencies and percentages.

Comparisons among spirometric phenotype groups were performed using one-way analysis of variance (ANOVA) for normally distributed continuous variables, Kruskal–Wallis tests for non-normally distributed variables, and chi-square or Fisher’s exact tests for categorical variables, as appropriate.

To identify variables associated with postoperative ventilatory burden, separate univariable linear regression models were fitted using natural-log-transformed VBI as the dependent variable and HC3 heteroskedasticity-robust standard errors. Variables meeting the predefined screening criterion of *p* < 0.10, together with variables retained because of their a priori clinical or study-specific relevance, were entered into the multivariable linear regression model. The multivariable model likewise used natural-log-transformed VBI and HC3 robust standard errors. Model specification, collinearity, residual behavior, and influential observations were evaluated before interpretation. Variance inflation factors were examined to assess multicollinearity.

All statistical tests were two-sided, and *p* values < 0.05 were considered statistically significant. No adjustment for multiple comparisons was performed because subgroup analyses were considered exploratory and interpreted as hypothesis-generating.

To facilitate transparency and reproducibility, the complete anonymized study dataset used for all statistical analyses, including the variables used to derive the VBI, is provided as Supplementary Table S1.

## 3. Results

### 3.1. Baseline Characteristics

A total of 129 patients undergoing elective cardiac surgery were included in the final study cohort (Figure 1). Preoperative spirometry was normal in 82 patients (63.6%), while 25 patients (19.4%) demonstrated an obstructive spirometric pattern, 20 patients (15.5%) demonstrated a restrictive spirometric pattern, and 2 patients (1.6%) demonstrated a mixed spirometric pattern. Because only two patients exhibited a mixed spirometric pattern, this group was excluded from subsequent phenotype-based analyses.

The cohort had a mean age of 65.1 ± 8.6 years and was predominantly male (71.3%). The mean body mass index was 29.4 ± 4.9 kg/m^2^, and 54.3% of patients were current or former smokers. The mean left ventricular ejection fraction (LVEF) was 47.2 ± 7.5%. The most frequently performed procedures were isolated valve surgery (42.6%) and isolated coronary artery bypass grafting (CABG) (33.3%), followed by combined/complex cardiac procedures (17.8%) and aortic/root surgery (6.2%).

Baseline characteristics stratified according to preoperative spirometric phenotype are presented in Table 1. No statistically significant differences were observed among the normal spirometry, restrictive spirometric pattern, and obstructive spirometric pattern groups with respect to demographic characteristics, smoking exposure, LVEF, operative variables, or surgery type distribution (all *p* > 0.05), indicating that the study groups were broadly comparable at baseline.

### 3.2. Primary Endpoint: Ventilatory Burden Index

**Figure 2:**
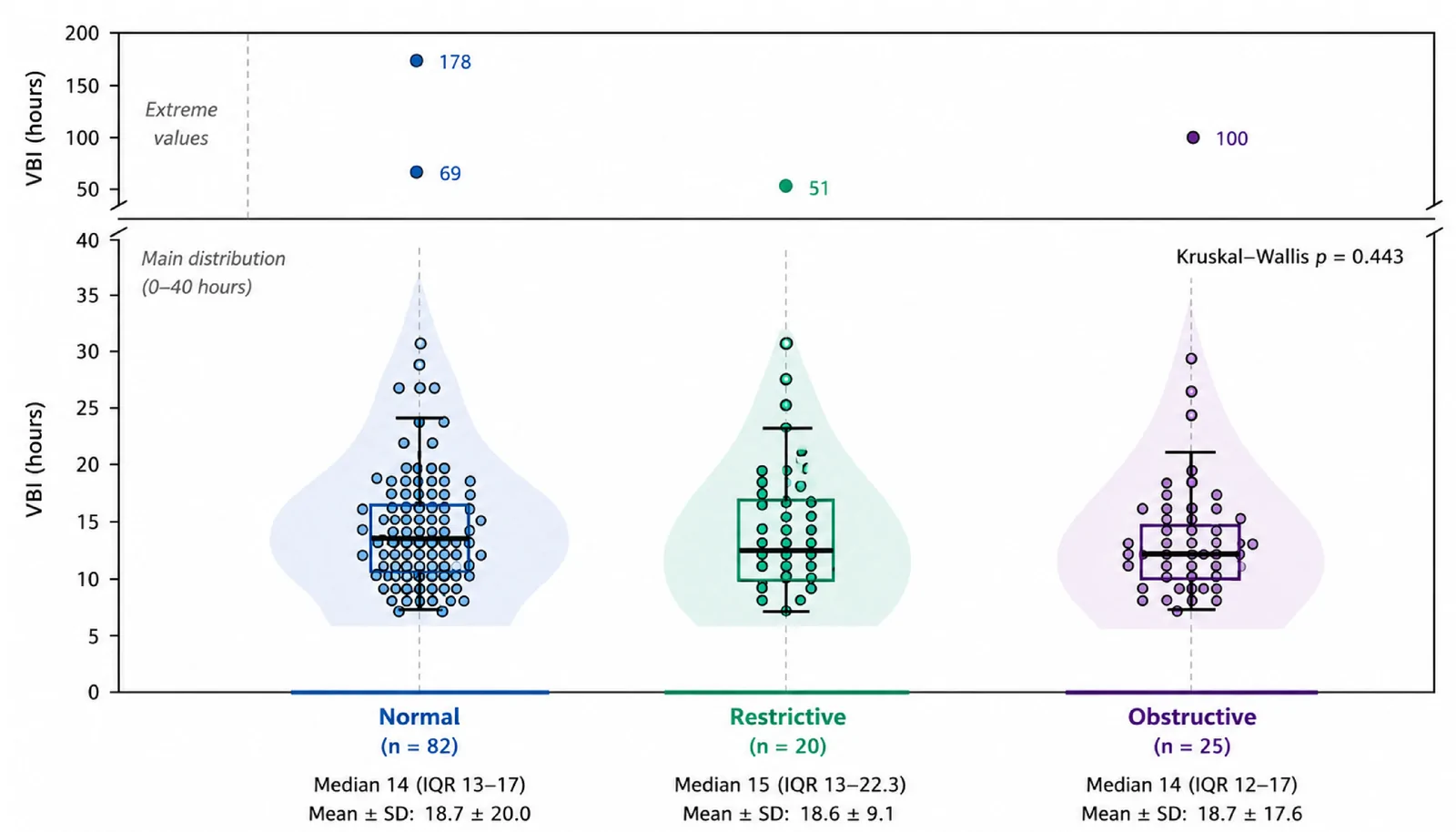
Ventilatory Burden Index according to preoperative spirometric phenotype. Raincloud plots showing the distribution of postoperative VBI among patients with normal spirometry (*n* = 82), a restrictive spirometric pattern (*n* = 20), and an obstructive spirometric pattern (*n* = 25). The lower panel displays VBI values between 0 and 40 h, whereas the upper panel displays extreme observations using a broken y-axis. Dots represent individual observations, and boxplots indicate the median and interquartile range (IQR). Differences among groups were assessed using the Kruskal–Wallis test (*p* = 0.443).

The primary endpoint analysis included 127 patients, after exclusion of the two patients with a mixed spirometric pattern from phenotype-based comparisons. VBI values according to preoperative spirometric phenotype are summarized in Table 2 and illustrated in Figure 2.

Median VBI values were similar across the three spirometric phenotype groups, measuring 14 h (IQR 13–17) in patients with normal spirometry, 15 h (IQR 13–22.3) in those with a restrictive spirometric pattern, and 14 h (IQR 12–17) in those with an obstructive spirometric pattern. Although patients with a restrictive spirometric pattern demonstrated a somewhat wider distribution of VBI values, substantial overlap was observed among all groups (Figure 2).

Comparison of VBI across preoperative spirometric phenotypes using the Kruskal–Wallis test demonstrated no statistically significant difference (H = 1.63, *p* = 0.443). The estimated effect size was small (η^2^ = 0.013), indicating that preoperative spirometric phenotype accounted for only a small proportion of the observed variability in VBI.

Overall, no statistically significant association was observed between preoperative spirometric phenotype and cumulative postoperative respiratory-support burden in this cohort. Although the estimated effect size was small, these findings should be interpreted as indicating the absence of a statistically demonstrable association within the present study rather than evidence that preoperative spirometric phenotype has no influence on postoperative respiratory recovery.

### 3.3. Secondary Respiratory Outcomes

Secondary respiratory outcomes according to preoperative spirometric phenotype are summarized in Table 2. Median invasive respiratory-support duration was 14 h (IQR 13–16) among patients with normal spirometry, 15 h (IQR 13–18.3) among those with a restrictive spirometric pattern, and 14 h (IQR 12–15) among those with an obstructive spirometric pattern. Although patients with a restrictive spirometric pattern demonstrated numerically longer invasive respiratory-support durations, the overall difference did not reach statistical significance (Kruskal–Wallis test, *p* = 0.089).

Post-extubation CPAP/AIRVO support was required in 8 patients (9.8%) with normal spirometry, 1 patient (5.0%) with a restrictive spirometric pattern, and 4 patients (16.0%) with an obstructive spirometric pattern. No statistically significant association was observed between preoperative spirometric phenotype and the requirement for post-extubation CPAP/AIRVO support (Fisher’s exact test, *p* = 0.467).

Similarly, no statistically significant differences were observed in post-extubation CPAP/AIRVO duration among the spirometric phenotype groups. The median duration was 0 h (IQR 0–0) in all groups, reflecting that most patients did not require additional non-invasive respiratory support following extubation (*p* = 0.454).

Overall, no statistically significant associations were observed between preoperative spirometric phenotype and invasive respiratory-support duration, the requirement for post-extubation CPAP/AIRVO support, or the duration of post-extubation respiratory support.

### 3.4. Predictors of Postoperative Ventilatory Burden

To identify factors associated with postoperative respiratory-support burden beyond preoperative spirometric phenotype, univariable and multivariable linear regression analyses were performed using natural-log-transformed VBI as the dependent variable. Candidate predictors included demographic characteristics, pulmonary function status, cardiac functional parameters, operative variables, and surgery type. Variables demonstrating potential associations in the univariable analyses, together with clinically relevant variables specified a priori, were subsequently evaluated in an adjusted multivariable model.

#### 3.4.1. Univariable Analysis

Separate univariable linear regression models were fitted using natural-log-transformed VBI as the dependent variable with HC3 heteroskedasticity-robust standard errors. The results are summarized in Table 3.

Older age was significantly associated with increased postoperative ventilatory burden, with each additional year corresponding to an approximately 1.0% increase in VBI (β = 0.0098 on the natural-log scale; 95% CI 0.0021–0.0175; *p* = 0.013). Conversely, higher LVEF was significantly associated with lower postoperative ventilatory burden, with each 1% increase in LVEF corresponding to an approximately 1.6% decrease in VBI (β = −0.0160 on the natural-log scale; 95% CI −0.0280 to −0.0040; *p* = 0.009).

Surgery type demonstrated a statistically significant global association with VBI (*p* = 0.018). Relative to aorta/root surgery, combined/complex procedures were associated with higher log-transformed VBI (β = 0.2496, 95% CI 0.0859–0.4133; *p* = 0.003), whereas the estimates for CABG and valve surgery did not reach statistical significance.

No statistically significant associations were observed for sex, BMI, smoking status, cumulative smoking exposure, cardiopulmonary bypass time, ischemic time, reperfusion time, or preoperative spirometric phenotype. Likewise, preoperative spirometric phenotype was not associated with VBI either in the global analysis (*p* = 0.751) or when restrictive and obstructive spirometric patterns were individually compared with normal spirometry.

Based on the predefined selection criterion (*p* < 0.10), age, LVEF, and surgery type were considered for multivariable analysis. BMI, cardiopulmonary bypass time, and preoperative spirometric phenotype were additionally retained because of their predefined clinical and study-specific relevance.

#### 3.4.2. Multivariable Analysis

**Figure 3:**
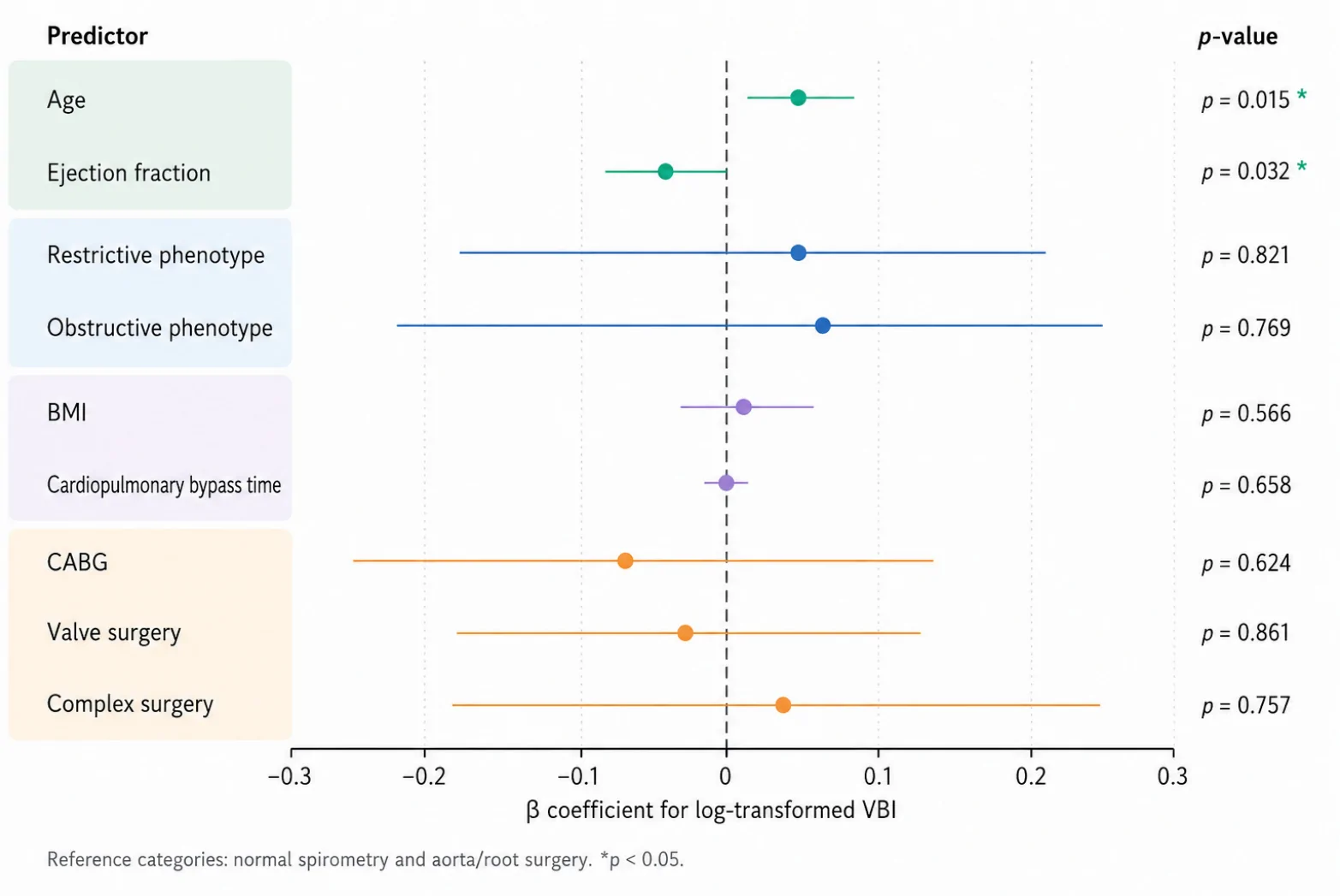
Multivariable linear regression model for postoperative Ventilatory Burden Index. Forest plot showing β coefficients and 95% confidence intervals for the variables included in the final multivariable linear regression model with natural-log-transformed VBI as the dependent variable. The vertical dashed line represents the null effect (β = 0). Reference categories were normal spirometry and aorta/root surgery.

Because cardiopulmonary bypass time and ischemic time demonstrated substantial collinearity, only cardiopulmonary bypass time was retained as the operative-duration variable in the final model. The multivariable linear regression model included preoperative spirometric phenotype, age, BMI, LVEF, surgery type, and cardiopulmonary bypass time. Results are presented in Table 4 and Figure 3.

After adjustment, no statistically significant associations were observed between postoperative VBI and preoperative spirometric phenotype, surgery type, BMI, or cardiopulmonary bypass time. Likewise, neither a restrictive nor an obstructive spirometric pattern was significantly associated with VBI relative to normal spirometry.

Older age was significantly associated with increased postoperative ventilatory burden (β = 0.0095, 95% CI 0.002 to 0.017; *p* = 0.015), whereas higher LVEF was significantly associated with reduced postoperative ventilatory burden (β = −0.0161, 95% CI −0.031 to −0.001; *p* = 0.032).

Within the final multivariable model, age and LVEF were the only variables demonstrating statistically significant associations with postoperative VBI. No statistically significant association was observed between broad preoperative spirometric phenotype and cumulative postoperative respiratory-support burden after adjustment for demographic, operative, and cardiac functional variables.

### 3.5. Exploratory Analysis of Ventilation Components

Exploratory analyses were performed to examine the individual components of postoperative respiratory support according to preoperative spirometric phenotype. The results are summarized in Supplementary Table S2.

Among the analyzed respiratory-support components, the largest numerical differences were observed for invasive respiratory-support duration. Patients with a restrictive spirometric pattern demonstrated numerically longer durations of both IPPV and total invasive respiratory support than patients with normal spirometry or an obstructive spirometric pattern. However, neither comparison reached statistical significance (IPPV: *p* = 0.089; total invasive respiratory support: *p* = 0.089).

In contrast, no statistically significant differences were observed in post-extubation respiratory-support duration among the spirometric phenotype groups. The numerical differences observed across phenotypes were therefore confined primarily to the invasive phase of postoperative respiratory support.

Taken together, these exploratory findings indicate a numerical tendency toward longer invasive respiratory-support requirements among patients with a restrictive spirometric pattern. Because these analyses did not reach statistical significance and were exploratory in nature, they should be interpreted cautiously and regarded as hypothesis-generating.

### 3.6. Spirometry Severity Status Analysis

Because broad preoperative spirometric phenotypes were not significantly associated with postoperative ventilatory burden, an exploratory analysis was performed using the original spirometric severity categories to evaluate whether postoperative respiratory-support requirements differed according to the severity of preoperative spirometric abnormalities. Total invasive respiratory-support duration was selected as the primary exploratory outcome because the preceding component analysis indicated that the largest numerical differences among spirometric phenotypes were observed during the invasive phase of postoperative respiratory support.

**Figure 4:**
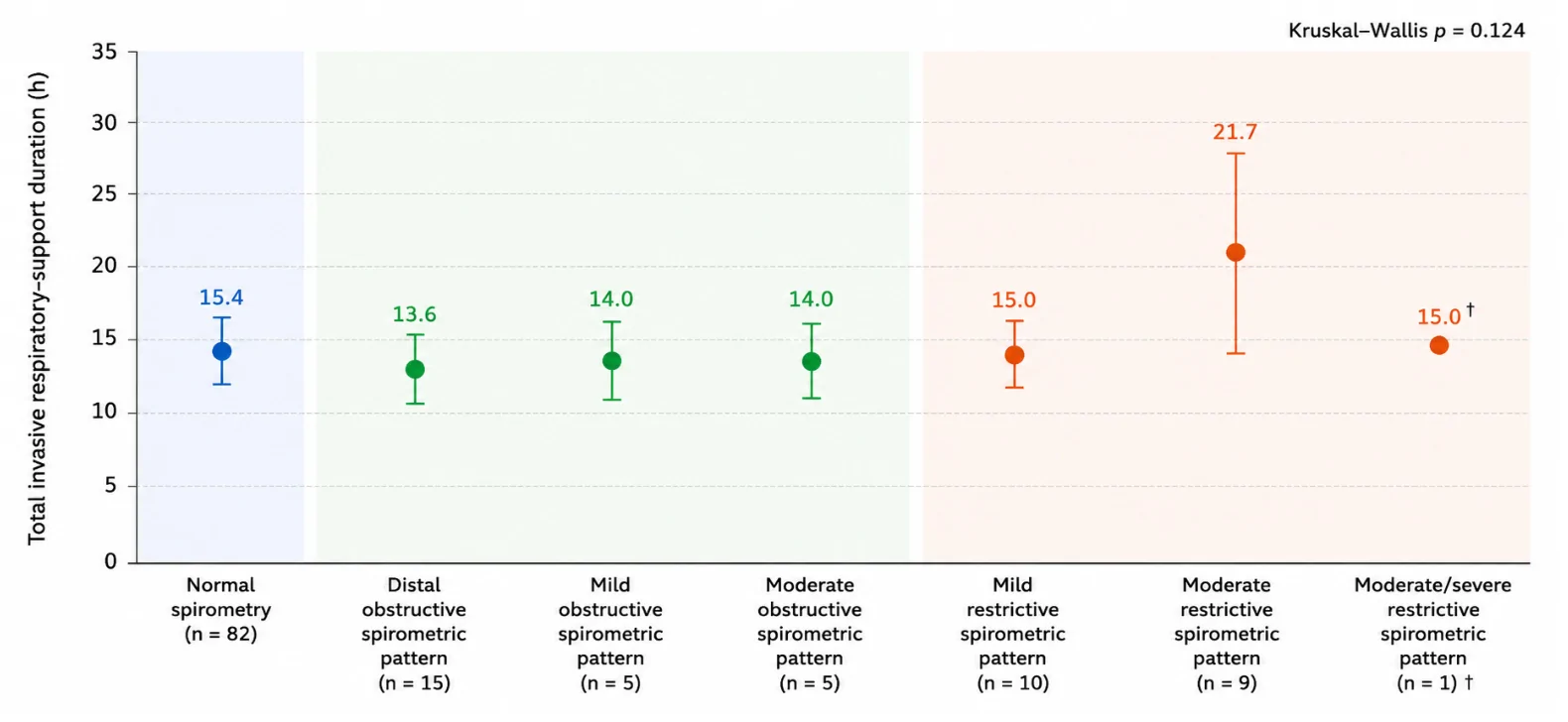
Total invasive respiratory-support duration according to preoperative spirometric severity status. Points represent the mean total invasive respiratory-support duration, defined as the cumulative duration of IPPV, synchronized intermittent mandatory ventilation (SIMV), and invasive CPAP. Whiskers indicate 95% confidence intervals. Sample sizes for each spirometric severity category are shown on the x-axis. Colors indicate spirometric phenotype: blue represents normal spirometry, green represents obstructive spirometric patterns, and orange represents restrictive spirometric patterns. The moderate/severe restrictive spirometric pattern category contained a single patient (*n* = 1) and is presented descriptively without a confidence interval. † Indicates the single-patient category, for which a confidence interval could not be calculated. Overall group differences were assessed using the Kruskal–Wallis test (*p* = 0.124).

Total invasive respiratory-support duration according to preoperative spirometric severity category is summarized in Supplementary Table S3 and illustrated in Figure 4. Although the overall comparison did not reach statistical significance (Kruskal–Wallis test, *p* = 0.124), patients with a moderate restrictive spirometric pattern demonstrated the longest total invasive respiratory-support duration (mean 21.7 h, median 16 h). In contrast, patients with distal obstructive, mild obstructive, moderate obstructive, and mild restrictive spirometric patterns demonstrated total invasive respiratory-support durations that were broadly comparable with those observed in patients with normal spirometry.

To further characterize these findings, the individual components of invasive respiratory support were analyzed according to spirometric severity category (Supplementary Table S2). Among these components, IPPV demonstrated the largest numerical differences across severity categories (Kruskal–Wallis test, *p* = 0.067), whereas no statistically significant differences were observed for SIMV (*p* = 0.359) or invasive CPAP duration (*p* = 0.845).

Overall, these exploratory analyses demonstrated a numerical tendency toward longer invasive respiratory-support requirements among patients with a moderate restrictive spirometric pattern. However, because the overall comparisons were not statistically significant and several severity categories contained very small sample sizes (Figure 4), these findings should be interpreted cautiously and regarded as hypothesis-generating rather than confirmatory.

## 4. Discussion

### 4.1. Principal Findings

In this prospective cohort of patients undergoing elective cardiac surgery, no statistically significant association was observed between broad preoperative spirometric phenotype and postoperative VBI, nor was preoperative spirometric phenotype significantly associated with VBI after adjustment for demographic, cardiac, and operative variables. Patients with normal spirometry, a restrictive spirometric pattern, and an obstructive spirometric pattern demonstrated comparable overall respiratory-support requirements, suggesting that broad preoperative spirometric phenotype alone may have limited ability to discriminate cumulative postoperative respiratory-support burden.

Although the primary endpoint was not significantly associated with preoperative spirometric phenotype, the exploratory analyses identified a numerical tendency toward longer invasive respiratory-support duration among patients with a restrictive spirometric pattern. This tendency was most evident when the individual components of respiratory support were examined separately, where patients with a restrictive spirometric pattern demonstrated numerically longer invasive respiratory-support duration than those with normal spirometry or an obstructive spirometric pattern. Likewise, the exploratory severity-category analysis showed that patients with a moderate restrictive spirometric pattern had the longest invasive respiratory-support duration. However, these findings did not reach statistical significance and should be interpreted cautiously in light of the limited sample sizes within several severity categories.

In the multivariable analysis, older age was associated with increased VBI, whereas higher LVEF was associated with lower VBI. No statistically significant associations were observed for preoperative spirometric phenotype, surgery type, BMI, or cardiopulmonary bypass time after adjustment for the other variables included in the model.

Taken together, the present findings suggest that broad preoperative spirometric phenotype alone may have limited ability to identify patients at increased risk of greater cumulative postoperative respiratory-support burden. Nevertheless, the exploratory analyses raise the possibility that patients with more pronounced restrictive spirometric abnormalities may experience longer invasive respiratory-support duration. Because these observations were exploratory and based on small subgroup sizes, they should be regarded as hypothesis-generating and require confirmation in larger prospective studies.

### 4.2. Broad Spirometric Phenotype and Global Respiratory Burden

Contrary to our initial hypothesis, broad preoperative spirometric phenotype was not significantly associated with postoperative VBI. Patients with normal spirometry, a restrictive spirometric pattern, and an obstructive spirometric pattern demonstrated comparable overall respiratory-support requirements, and preoperative spirometric phenotype was not significantly associated with VBI after multivariable adjustment. These findings suggest that broad preoperative spirometric phenotype alone may not fully capture the complex physiological determinants of postoperative respiratory recovery following cardiac surgery.

Several factors may explain the absence of a statistically significant relationship between preoperative spirometric phenotype and VBI. VBI was designed as a study-specific, unweighted composite integrating the cumulative duration of IPPV, SIMV, invasive CPAP, and clinically indicated post-extubation CPAP/AIRVO support, thereby capturing respiratory-support requirements across both pre- and post-extubation phases rather than relying on a single recovery milestone such as extubation time. Although this approach captures the overall burden of invasive respiratory support, it also reflects multiple clinical processes that extend beyond baseline pulmonary mechanics. Age, cardiac function, intraoperative management, postoperative hemodynamics, fluid balance, analgesia, sedation practices, and institutional extubation protocols may all contribute to cumulative respiratory-support duration.

The present findings also highlight an important distinction between baseline pulmonary function and postoperative respiratory burden. Cardiac surgery introduces multiple non-pulmonary determinants of respiratory recovery, including transient myocardial dysfunction, fluid shifts, diaphragmatic impairment, postoperative pain, and perioperative physiological stress. In this setting, the contribution of broad preoperative spirometric abnormalities to cumulative postoperative respiratory-support burden may be relatively modest when compared with the combined effects of these perioperative factors [19].

Importantly, the absence of a statistically significant association between preoperative spirometric phenotype and VBI should not be interpreted as evidence that pulmonary function is clinically unimportant in patients undergoing cardiac surgery. Rather, the present findings suggest that broad spirometric phenotype alone may not be sufficiently sensitive to identify specific aspects of postoperative respiratory recovery. In the exploratory analyses, patients with a restrictive spirometric pattern demonstrated a numerical tendency toward longer invasive respiratory-support duration. However, these observations did not reach statistical significance and should be interpreted cautiously pending confirmation in larger prospective studies.

### 4.3. Restrictive Spirometric Pattern and Invasive Respiratory Recovery

Although broad preoperative spirometric phenotype was not significantly associated with overall postoperative VBI, the exploratory analyses demonstrated a numerical tendency toward longer invasive respiratory-support duration among patients with a restrictive spirometric pattern. This tendency was most apparent when the individual components of postoperative respiratory support were examined separately. Patients with a restrictive spirometric pattern demonstrated numerically longer invasive respiratory-support duration, and the exploratory severity-category analysis showed that patients with a moderate restrictive spirometric pattern had the longest invasive respiratory-support duration.

These observations are biologically plausible. A restrictive spirometric pattern is characterized by reduced FVC with preservation of the FEV_1_/FVC ratio and may reflect reduced ventilatory capacity, although confirmation of true restrictive ventilatory impairment requires lung-volume measurements [20]. Following cardiac surgery, respiratory function is challenged by sternotomy-related pain, diaphragmatic dysfunction, atelectasis, pleural alterations, fluid shifts, and the inflammatory effects of cardiopulmonary bypass [21]. Under these conditions, patients with reduced pulmonary reserve may require longer periods of invasive respiratory support before achieving stable spontaneous respiration [9,22].

The exploratory findings of the present study suggest that any potential effect of a restrictive spirometric pattern may be confined primarily to the invasive phase of postoperative respiratory support rather than to cumulative postoperative respiratory-support burden. The largest numerical differences were observed for invasive respiratory-support duration, whereas little difference was observed in post-extubation respiratory-support requirements. From a physiological perspective, successful liberation from invasive mechanical ventilation requires adequate respiratory muscle performance, sufficient lung recruitment, and maintenance of effective gas exchange without mechanical assistance. Patients with reduced pulmonary reserve may therefore require a longer period to achieve these conditions, even if their subsequent postoperative recovery is comparable with that of patients in the other spirometric groups.

These observations are also broadly consistent with previous reports suggesting that impaired pulmonary reserve may be associated with delayed postoperative respiratory recovery [6,9,22]. Whereas obstructive spirometric abnormalities primarily reflect airflow limitation, a restrictive spirometric pattern may indicate reduced ventilatory capacity. Consequently, a restrictive spirometric pattern could become more relevant during periods of increased respiratory demand, such as the early postoperative period following cardiac surgery. However, the present study did not demonstrate statistically significant differences in the primary endpoint, and these physiological considerations should therefore be regarded as explanatory rather than confirmatory.

The exploratory severity-category analyses included relatively small subgroups, limiting statistical power and precluding definitive conclusions. Consequently, these findings should be interpreted cautiously and regarded as hypothesis-generating. Future studies incorporating comprehensive pulmonary-function assessment, including lung-volume measurements, diffusion-capacity testing, and respiratory muscle evaluation, will be necessary to determine whether specific physiological characteristics associated with a restrictive spirometric pattern improve prediction of postoperative ventilator liberation following cardiac surgery.

### 4.4. Comparison with Previous Literature

The relationship between preoperative pulmonary function and postoperative outcomes following cardiac surgery has been investigated for several decades; however, the literature remains heterogeneous with respect to both pulmonary assessment methods and clinical endpoints. Most previous studies have focused on traditional postoperative pulmonary complications, prolonged mechanical ventilation, hospital length of stay, or mortality rather than on integrated measures of respiratory-support burden [23,24]. Consequently, direct comparisons with the present study should be made cautiously.

Previous investigations have generally evaluated individual spirometric parameters, such as FEV_1_ or FVC, rather than broad preoperative spirometric phenotypes, making direct comparison with the present analysis challenging [25,26,27].

Several studies have reported associations between impaired preoperative pulmonary function and adverse postoperative outcomes, particularly among patients with restrictive spirometric abnormalities or markedly reduced pulmonary-function parameters. Reduced FVC and other markers of impaired pulmonary reserve have been associated with prolonged mechanical ventilation, increased pulmonary complications, and longer intensive care unit stays following cardiac surgery. These observations support the concept that baseline respiratory capacity may influence postoperative recovery, particularly during the period immediately following liberation from invasive mechanical ventilation [6,28,29].

The findings of the present study are broadly consistent with this body of literature while also highlighting important differences. Although broad preoperative spirometric phenotype was not significantly associated with VBI, the exploratory analyses demonstrated a numerical tendency toward longer invasive respiratory-support duration among patients with a restrictive spirometric pattern. These observations raise the possibility that the influence of pulmonary reserve may be more evident during specific phases of postoperative respiratory recovery than when assessed using an integrated composite outcome such as VBI. This may partly explain why previous studies evaluating different respiratory endpoints have reported heterogeneous results regarding the prognostic value of preoperative spirometry.

The present findings also support the concept that restrictive and obstructive spirometric abnormalities may have different perioperative implications. Whereas obstructive spirometric patterns primarily reflect airflow limitation, restrictive spirometric patterns may indicate reduced ventilatory capacity and pulmonary reserve. During the early postoperative period, respiratory function is challenged by sternotomy, postoperative pain, diaphragmatic dysfunction, fluid shifts, and atelectatic changes. Under these circumstances, reduced pulmonary reserve may contribute to prolonged invasive respiratory-support requirements. Although this interpretation is biologically plausible, it should be considered exploratory because the observed differences did not reach statistical significance.

At the same time, our findings differ from studies reporting strong independent associations between abnormal spirometry and major postoperative outcomes. One possible explanation is that broad spirometric phenotype may not fully characterize pulmonary reserve. Standard spirometry provides limited information regarding lung volumes, gas-exchange capacity, respiratory muscle performance, and regional lung mechanics, all of which may contribute to postoperative respiratory recovery. Consequently, more comprehensive physiological assessment may be required to better define the relationship between preoperative pulmonary status and postoperative ventilatory outcomes.

Taken together, the available evidence and the findings of the present study suggest that pulmonary reserve remains an important component of postoperative respiratory recovery. However, its contribution may be more nuanced than previously appreciated and may differ according to the respiratory endpoint evaluated. Larger prospective studies incorporating comprehensive pulmonary-function assessment will be necessary to clarify the relationship between preoperative spirometric abnormalities and specific phases of postoperative respiratory recovery.

These findings also have implications for contemporary perioperative risk assessment. Widely used cardiac surgical risk models, including EuroSCORE II and the Society of Thoracic Surgeons (STS) Risk Score [30,31], recognize chronic pulmonary disease as an important determinant of postoperative morbidity and mortality. However, these models rely primarily on the presence of clinically diagnosed pulmonary disease rather than objective spirometric phenotypes. The present findings suggest that broad preoperative spirometric phenotype alone may not provide substantial incremental information regarding cumulative postoperative respiratory-support burden beyond established clinical risk factors. Nevertheless, the exploratory observations regarding moderate restrictive spirometric patterns indicate that more detailed physiological assessment may warrant further investigation in future refinements of perioperative respiratory risk stratification.

### 4.5. Age, Cardiac Function, and Postoperative Ventilatory Burden

In the adjusted multivariable model, age and LVEF were the only variables that remained significantly associated with postoperative VBI, whereas preoperative spirometric phenotype, operative variables, and surgery type were not significantly associated with VBI after adjustment. These findings suggest that postoperative respiratory-support requirements reflect the combined influence of multiple demographic, cardiac, and perioperative factors rather than preoperative spirometric phenotype alone.

The association between advancing age and increased VBI is biologically plausible. Aging is accompanied by progressive reductions in respiratory muscle strength, chest-wall compliance, pulmonary elastic recoil, and physiological reserve [32]. In addition, older patients may have a diminished capacity to compensate for the respiratory stress imposed by cardiopulmonary bypass, postoperative pain, fluid shifts, and prolonged bed rest. Collectively, these factors may delay recovery of effective spontaneous ventilation and prolong postoperative respiratory-support requirements following cardiac surgery.

Similarly, the inverse association between LVEF and VBI highlights the close interaction between cardiac and pulmonary function during postoperative recovery. Reduced left ventricular systolic function may contribute to elevated pulmonary venous pressures, impaired pulmonary fluid clearance, and reduced cardiopulmonary reserve. Even in the absence of overt heart failure, patients with lower LVEF may require longer periods of respiratory support to maintain adequate oxygen delivery and hemodynamic stability during the early postoperative period [33,34].

These observations emphasize that postoperative respiratory recovery following cardiac surgery is influenced by multiple physiological systems. Although spirometry provides valuable information regarding baseline ventilatory function, postoperative respiratory-support requirements are also affected by age, cardiac performance, operative factors, and perioperative management. The present findings therefore support a multidimensional approach to understanding postoperative respiratory recovery rather than one based solely on preoperative spirometric phenotype.

From a clinical perspective, these findings support the integration of pulmonary and cardiovascular assessment within perioperative risk evaluation. Patients of advanced age or with lower LVEF may warrant closer postoperative respiratory monitoring and individualized ventilatory-management strategies. However, these observations should be interpreted within the context of this observational study and require confirmation in larger prospective cohorts before influencing routine clinical decision-making.

### 4.6. Clinical Implications

The present findings have several implications for perioperative respiratory assessment in cardiac surgery. Spirometry is routinely used to identify patients with underlying ventilatory abnormalities and may contribute to decisions regarding pulmonary optimization, perioperative monitoring, and postoperative respiratory management. However, the present results suggest that broad preoperative spirometric phenotype alone may have limited ability to identify patients at increased risk of greater postoperative VBI. Patients with normal spirometry, a restrictive spirometric pattern, and an obstructive spirometric pattern demonstrated comparable VBI values, indicating that broad spirometric categorization alone may not fully capture the complexity of postoperative respiratory recovery.

The exploratory analyses nevertheless provide additional context. Although a restrictive spirometric pattern was not significantly associated with the primary endpoint, patients with a moderate restrictive spirometric pattern demonstrated numerically longer invasive respiratory-support duration than the other severity categories. Because these observations were exploratory and based on small subgroup sizes, they should not be considered sufficient to guide clinical decision-making. However, they suggest that greater attention to patients with more pronounced restrictive spirometric abnormalities may be warranted in future studies evaluating postoperative ventilator liberation.

The present findings also emphasize the importance of integrating pulmonary assessment within a broader perioperative risk evaluation. In the adjusted analyses, age and LVEF remained significantly associated with VBI, whereas broad preoperative spirometric phenotype did not. These findings suggest that postoperative respiratory-support requirements reflect the combined influence of pulmonary, cardiac, and perioperative factors rather than spirometric phenotype alone.

From a practical perspective, perioperative respiratory-risk assessment may therefore benefit from combining spirometric findings with established clinical characteristics, including age, cardiac function, and other patient-specific factors, rather than relying on broad spirometric phenotype alone. Such an approach may help identify patients who could benefit from closer postoperative respiratory monitoring and individualized ventilatory-management strategies.

Although the present findings do not support the use of broad preoperative spirometric phenotype as a standalone predictor of postoperative VBI, the exploratory analyses suggest that the severity of spirometric abnormalities, particularly a moderate restrictive spirometric pattern, merits further investigation. Confirmation in larger prospective studies incorporating comprehensive pulmonary-function assessment will be necessary before these observations can be translated into routine clinical practice.

### 4.7. Strengths and Limitations

This study has several strengths. The prospective design enabled standardized preoperative spirometric assessment and systematic collection of postoperative respiratory-support outcomes in a well-characterized cohort of patients undergoing elective cardiac surgery. The use of VBI, a composite measure integrating the cumulative duration of IPPV, SIMV, and invasive CPAP, provided a comprehensive assessment of cumulative postoperative respiratory-support burden across invasive and post-extubation phases. In addition, complementary analyses of individual respiratory-support components and spirometric severity categories offered further insight into specific phases of postoperative respiratory recovery. Finally, multivariable modeling enabled simultaneous evaluation of preoperative spirometric phenotype alongside demographic, cardiac, and operative variables.

Several limitations should be acknowledged. First, this was a single-center secondary analysis of a prospectively collected cohort, which may limit the generalizability of the findings to other cardiac surgical populations and perioperative management protocols. Second, the sample size, particularly the restrictive (*n* = 20) and obstructive (*n* = 25) groups, limited the precision and statistical power of phenotype-based comparisons. Consequently, clinically meaningful differences cannot be excluded despite the absence of statistically significant associations. This limitation was particularly relevant for the exploratory severity-category analyses, in which several categories contained only a small number of patients and should therefore be interpreted as hypothesis-generating.

Third, classification of restrictive spirometric patterns was based exclusively on spirometry. Because lung-volume measurements were not available, true restrictive ventilatory impairment could not be confirmed. Similarly, diffusion-capacity testing, respiratory muscle assessment, and imaging-based evaluation of pulmonary structure and function were not performed. Consequently, important aspects of pulmonary reserve may not have been fully characterized.

Fourth, as with any observational secondary analysis, residual confounding cannot be excluded. Variables such as intraoperative fluid balance, postoperative analgesic strategies, diaphragmatic function, rehabilitation practices, and other aspects of perioperative management were not included in the present analyses and may also influence postoperative respiratory recovery.

Finally, VBI is a study-specific, unweighted composite developed to quantify cumulative postoperative respiratory-support duration across invasive and post-extubation phases. Although this approach captures a broader respiratory-support trajectory than isolated measures such as extubation time, VBI has not undergone external validation, and equal weighting of support modalities does not account for differences in their physiological intensity. This construction may also reduce sensitivity to effects confined to a specific phase of respiratory recovery.

Despite these limitations, the primary, multivariable, ventilation-component, and severity-category analyses demonstrated a consistent overall pattern. Broad preoperative spirometric phenotype was not significantly associated with postoperative VBI, whereas the exploratory analyses identified a numerical tendency toward longer invasive respiratory-support duration among patients with more pronounced restrictive spirometric abnormalities. These observations provide a rationale for larger multicenter studies incorporating comprehensive pulmonary-function assessment to further evaluate the relationship between preoperative pulmonary status and postoperative respiratory recovery following cardiac surgery.

**Figure 5:**
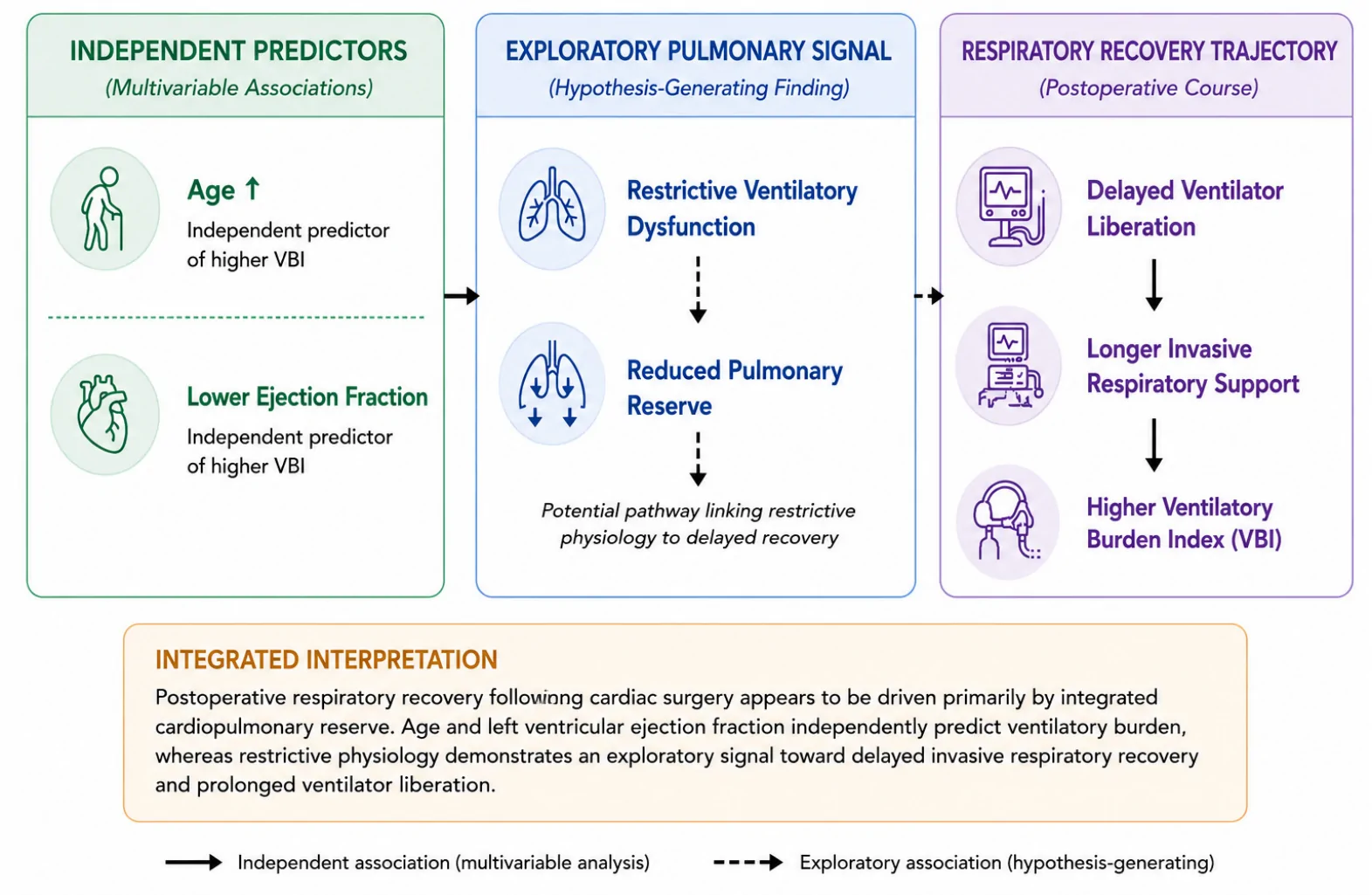
Hypothesized conceptual framework linking preoperative spirometric phenotype, cardiac function, and postoperative respiratory recovery following cardiac surgery. Solid arrows represent statistically significant associations observed in the multivariable analysis, whereas dashed arrows represent exploratory observations requiring further validation. The framework summarizes the hypothesized relationships among preoperative spirometric phenotype, cardiac function, perioperative factors, and postoperative respiratory recovery proposed by the present study.

A conceptual framework summarizing the proposed relationships between preoperative spirometric phenotype, cardiac function, and postoperative respiratory recovery is presented in Figure 5.

## 5. Conclusions

In this prospective cohort of patients undergoing elective cardiac surgery, no statistically significant association was observed between broad preoperative spirometric phenotype and postoperative VBI, and preoperative spirometric phenotype was not significantly associated with VBI after multivariable adjustment. These findings suggest that broad preoperative spirometric phenotype alone may have limited ability to identify patients at increased risk of greater cumulative postoperative respiratory-support burden.

The exploratory analyses demonstrated a numerical tendency toward longer invasive respiratory-support duration among patients with a restrictive spirometric pattern, particularly within the moderate severity category. However, these observations did not reach statistical significance and should be regarded as hypothesis-generating because of the limited subgroup sizes.

In the adjusted analyses, age was associated with increased VBI, whereas higher LVEF was associated with lower VBI. These findings suggest that postoperative respiratory-support requirements reflect the combined influence of demographic, cardiac, and perioperative factors in addition to baseline pulmonary function.

Overall, the present study does not support the use of broad preoperative spirometric phenotype as a standalone predictor of cumulative postoperative respiratory-support burden following cardiac surgery. Nevertheless, the exploratory findings warrant further investigation in larger prospective multicenter studies incorporating comprehensive pulmonary-function assessment to determine whether specific spirometric abnormalities are associated with delayed liberation from invasive mechanical ventilation.

## Figures and Tables

**Table 1:** Baseline characteristics of patients according to preoperative spirometric phenotype.

Variable	Normal (*n* = 82)	Restrictive (*n* = 20)	Obstructive (*n* = 25)	*p*-Value
Age, years	64.7 ± 9.5	66.7 ± 5.2	64.6 ± 7.8	0.803
Male sex, *n* (%)	62 (75.6)	12 (60.0)	17 (68.0)	0.344
BMI, kg/m^2^	29.1 ± 4.2	31.1 ± 5.2	29.1 ± 6.5	0.265
Smoking history, *n* (%)	42 (51.2)	12 (60.0)	16 (64.0)	0.474
Pack-years	13.7 ± 16.6	22.1 ± 26.1	19.0 ± 20.3	0.36
EF, %	46.6 ± 7.5	46.2 ± 9.9	49.4 ± 4.2	0.382
Surgery type, *n* (%)				0.124
CABG	31 (37.8)	4 (20.0)	8 (32.0)	
Valve	32 (39.0)	9 (45.0)	14 (56.0)	
Complex	13 (15.9)	7 (35.0)	3 (12.0)	
Aorta/Root Surgery	6 (7.3)	0 (0.0)	0 (0.0)	
Bypass time, min	107.3 ± 30.4	108.5 ± 27.0	95.1 ± 22.9	0.229
Ischemic time, min	67.9 ± 22.4	65.0 ± 15.0	59.2 ± 17.5	0.31
Reperfusion time, min	30.3 ± 12.1	35.0 ± 16.9	29.0 ± 12.4	0.488

**Table 2:** Postoperative respiratory outcomes according to preoperative spirometric phenotype. Data are presented as mean ± standard deviation, median (interquartile range), or *n* (%), as appropriate. Continuous variables were compared using the Kruskal–Wallis test and categorical variables using Fisher’s exact test.

Outcome	Normal (*n* = 82)	Restrictive (*n* = 20)	Obstructive (*n* = 25)	*p*-Value
Ventilatory Burden Index (h)				
Mean ± SD	18.7 ± 20.0	18.6 ± 9.1	18.7 ± 17.6	
Median (IQR)	14 (13–17)	15 (13–22.3)	14 (12–17)	0.443
Intubation Time (h)				
Mean ± SD	15.4 ± 4.5	18.0 ± 8.9	13.8 ± 2.4	
Median (IQR)	14 (13–16)	15 (13–18.3)	14 (12–15)	0.089
Post-extubation CPAP/AIRVO Requirement				
*n* (%)	8 (9.8)	1 (5.0)	4 (16.0)	0.467
Post-extubation CPAP/AIRVO Duration (h)				
Mean ± SD	3.4 ± 18.4	0.6 ± 2.7	5.0 ± 17.9	
Median (IQR)	0 (0–0)	0 (0–0)	0 (0–0)	0.454

**Table 3:** Univariable linear regression analyses of factors associated with postoperative VBI. Separate univariable linear regression models were fitted for each predictor using natural-log-transformed VBI as the dependent variable and HC3 heteroskedasticity-robust standard errors. β coefficients represent the estimated change in log(VBI) per one-unit increase in continuous predictors or relative to the specified reference category for categorical predictors. For categorical variables with multiple levels (spirometric phenotype and surgery type), the global *p*-value represents the overall effect of the variable, whereas individual β coefficients represent comparisons with the reference category. Normal spirometry and aorta/root surgery served as the reference categories. R^2^ and adjusted R^2^ are reported for each univariable model.

Predictor	β Coefficient	SE	95% CI	*p*-Value	R^2^	Adjusted R^2^
Age (per year)	0.0098	0.0039	0.0021 to 0.0175	0.013	0.039	0.032
Male sex vs. female	−0.092	0.1006	−0.2910 to 0.1071	0.362	0.01	0.002
BMI (per kg/m^2^)	0.0025	0.0094	−0.0162 to 0.0212	0.793	0.001	−0.007
Smoking history vs. none	−0.0358	0.0781	−0.1903 to 0.1187	0.647	0.002	−0.006
Pack-years (per pack-year)	0.0015	0.0032	−0.0048 to 0.0079	0.630	0.005	−0.003
LVEF (per %)	−0.016	0.006	−0.0280 to −0.0040	0.009	0.078	0.071
Cardiopulmonary bypass time (per min)	0.0009	0.0011	−0.0014 to 0.0031	0.443	0.003	−0.005
Ischemic time (per min)	0.0013	0.0014	−0.0014 to 0.0040	0.342	0.004	−0.004
Reperfusion time (per min)	0.0015	0.0035	−0.0055 to 0.0084	0.677	0.002	−0.006
Spirometric phenotype (global)	—	—	—	0.751	0.004	−0.012
└ Restrictive spirometric pattern vs. normal spirometry	0.0702	0.0976	−0.1231 to 0.2634	0.474	—	—
└ Obstructive spirometric pattern vs. normal spirometry	−0.0088	0.1069	−0.2203 to 0.2028	0.935	—	—
Surgery type (global)	—	—	—	0.018	0.019	−0.005
└ CABG vs. aorta/root surgery	0.1131	0.0863	−0.0577 to 0.2838	0.192	—	—
└ Combined/complex surgery vs. aorta/root surgery	0.2496	0.0827	0.0859 to 0.4133	0.003	—	—
└ Valve surgery vs. aorta/root surgery	0.1227	0.0682	−0.0123 to 0.2577	0.074	—	—

Note: The symbol “└” indicates individual category-level comparisons nested under the corresponding global categorical variable.

**Table 4:** Multivariable linear regression analysis of factors associated with postoperative VBI. Natural-log-transformed VBI was used as the dependent variable. β coefficients, robust standard errors (SE), 95% confidence intervals (CI), and *p*-values are shown. Reference categories were normal spirometry and aorta/root surgery. Model performance: R^2^ = 0.123; adjusted R^2^ = 0.056.

Predictor	β Coefficient	SE (HC3)	95% CI	*p*-Value
Spirometric phenotype (reference: normal spirometry)				
└ Restrictive spirometric pattern	0.02	0.09	−0.155 to 0.196	0.821
└ Obstructive spirometric pattern	0.031	0.107	−0.178 to 0.240	0.769
Surgery type (reference: aorta/root surgery)				
└ CABG	−0.051	0.105	−0.256 to 0.154	0.624
└ Combined/complex surgery	0.035	0.111	−0.184 to 0.252	0.757
└ Valve surgery	−0.014	0.079	−0.169 to 0.141	0.861
Age (per year)	0.0095	0.0039	0.002 to 0.017	0.015
BMI (per kg/m^2^)	0.0055	0.0096	−0.013 to 0.024	0.566
LVEF (per %)	−0.0161	0.0075	−0.031 to −0.001	0.032
Cardiopulmonary bypass time (per min)	−0.0006	0.0013	−0.003 to 0.002	0.658

Note: The symbol “└” indicates individual category-level comparisons nested under the corresponding global categorical variable.

## Data Availability

The anonymized patient-level dataset supporting the findings of this study is provided as Supplementary Table S1. Additional information is available from the corresponding author upon reasonable request.

## Supplementary Materials

The following supporting information can be downloaded at: https://www.mdpi.com/article/10.3390/biomedicines14081799/s1. Table S1. Anonymized patient-level dataset used for all analyses. Supplementary Table S2. Individual components of postoperative respiratory support according to preoperative spirometric phenotype. Data are presented as mean ± standard deviation, median (interquartile range), or n (%), as appropriate. Continuous variables were compared using the Kruskal–Wallis test. Abbreviations: IPPV, intermittent positive-pressure ventilation; SIMV, synchronized intermittent mandatory ventilation; CPAP, continuous positive airway pressure; AIRVO, high-flow nasal oxygen therapy; IOT, total invasive respiratory-support duration (IPPV + SIMV + invasive CPAP); IQR, interquartile range; SD, standard deviation. Supplementary Table S3. Total invasive respiratory-support duration according to spirometric severity status. Total invasive respiratory-support duration (IOT) according to spirometric severity status. Data are presented as mean ± standard deviation and median (interquartile range). Comparisons were performed using the Kruskal–Wallis test. Abbreviations: IOT, total invasive respiratory-support duration; IQR, interquartile range; SD, standard deviation.

## Abbreviations

**Abbreviation**	**Definition**
AIRVO	High-Flow Nasal Oxygen Therapy (AIRVO™ System)
ANOVA	Analysis of Variance
ATS	American Thoracic Society
BMI	Body Mass Index
CABG	Coronary Artery Bypass Grafting
CI	Confidence Interval
CPAP	Continuous Positive Airway Pressure
LVEF	Ejection Fraction
ERS	European Respiratory Society
FEV1	Forced Expiratory Volume in One Second
FVC	Forced Vital Capacity
IOT	Total Invasive Respiratory-Support Duration
IPPV	Intermittent Positive-Pressure Ventilation
IQR	Interquartile Range
SD	Standard Deviation
SIMV	Synchronized Intermittent Mandatory Ventilation
STROBE	Strengthening the Reporting of Observational Studies in Epidemiology
VBI	Ventilatory Burden Index
