# Supplementary material for: Pulmonary Reserve and Respiratory Recovery After Cardiac Surgery: Insights from Preoperative Spirometric Phenotyping
Source: Biomedicines. 2026 Aug 10;14(8):1799. doi: 10.3390/biomedicines14081799 (PMC13510278; doi:10.3390/biomedicines14081799)
Supplement: Supplementary file 1 [file biomedicines-14-01799-s001.zip › Table S2.pdf]

Outcome	Normal (n = 82)	Restrictive (n = 20)	Obstructive (n = 25)	p-value
**IPPV Duration (h)**				
Mean ± SD	5.63 ± 1.73	5.40 ± 1.60	4.80 ± 1.35	
Median (IQR)	6 (4–6)	5 (4–6)	5 (4–6)	**0.089**
**SIMV Duration (h)**				
Mean ± SD	8.04 ± 3.14	10.80 ± 8.42	7.52 ± 2.16	
Median (IQR)	7.5 (6–9)	8 (6–11.75)	7 (6–9)	**0.430**
**Invasive CPAP Duration (h)**				
Mean ± SD	1.70 ± 1.92	1.80 ± 1.11	1.44 ± 0.71	
Median (IQR)	1 (1–2)	1.5 (1–2.25)	1 (1–2)	**0.493**
**Total Invasive Respiratory Support (IOT) (h)**				
Mean ± SD	15.37 ± 4.45	18.00 ± 8.87	13.76 ± 2.35	
Median (IQR)	14 (13–16)	15 (13–18.25)	14 (12–15)	**0.089**
**Post-extubation CPAP/AIRVO Duration (h)**				
Mean ± SD	3.35 ± 18.38	0.60 ± 2.68	4.96 ± 17.94	
Median (IQR)	0 (0–0)	0 (0–0)	0 (0–0)	**0.454**

Supplementary Table S2. Individual components of postoperative respiratory support according to preoperative spirometric phenotype. Data are presented as mean ± standard deviation, median (interquartile range), or n (%), as appropriate. Continuous variables were compared using the Kruskal–Wallis test. Abbreviations: IPPV, intermittent positive-pressure ventilation; SIMV, synchronized intermittent mandatory ventilation; CPAP, continuous positive airway pressure; AIRVO, high-flow nasal oxygen therapy; IOT, total invasive respiratory-support duration (IPPV + SIMV + invasive CPAP); IQR, interquartile range; SD, standard deviation.
