# Supplementary material for: Pulmonary Reserve and Respiratory Recovery After Cardiac Surgery: Insights from Preoperative Spirometric Phenotyping
Source: Biomedicines. 2026 Aug 10;14(8):1799. doi: 10.3390/biomedicines14081799 (PMC13510278; doi:10.3390/biomedicines14081799)
Supplement: Supplementary file 1 [file biomedicines-14-01799-s001.zip › Table S3.pdf]

Spirometric Status	n	Mean IOT ± SD (h)	Median IOT (IQR) (h)
Normal	82	15.4 ± 4.5	14 (13–16)
Distal Obstructive Syndrome	15	13.6 ± 2.1	14 (12–15)
Mild Obstructive Dysfunction	5	14.0 ± 2.9	13 (12–14)
Moderate Obstructive Dysfunction	5	14.0 ± 2.9	13 (12–16)
Mild Restrictive Dysfunction	10	15.0 ± 3.9	14 (13–15)
Moderate Restrictive Dysfunction	9	21.7 ± 11.9	16 (15–23)
Moderate/Severe Restrictive Dysfunction	1	15.0	15
**Overall p-value**			**0.124**

Supplementary Table S3. Total invasive respiratory-support duration according to spirometric severity status. Total invasive respiratory-support duration (IOT) according to spirometric severity status. Data are presented as mean ± standard deviation and median (interquartile range). Comparisons were performed using the Kruskal–Wallis test. Abbreviations: IOT, total invasive respiratory-support duration; IQR, interquartile range; SD, standard deviation.
